# Extracorporeal Membrane Oxygenation for Severe Hypoxemia in Burn Patients: Analysis from Taiwan National Health Insurance Research Database

**DOI:** 10.3390/jcm14186623

**Published:** 2025-09-19

**Authors:** Jiun-Yu Lin, Yi-Ting Tsai, Chih-Yuan Lin, Hung-Yen Ke, Yi-Chang Lin, Jia-Lin Chen, Hsiang-Yu Yang, Chien-Ting Liu, Wu-Chien Chien, Chien-Sung Tsai, Po-Shun Hsu, Shih-Ying Sung

**Affiliations:** 1 Division of Cardiovascular Surgery, Department of Surgery, Tri-Service General Hospital, National Defense Medical University, Taipei 114, Taiwan; acidyulin@gmail.com (J.-Y.L.);; 2 Department of Anesthesia, Tri-Service General Hospital, National Defense Medical University, Taipei 114, Taiwan; 3 School of Public Health, National Defense Medical University, Taipei 114, Taiwan; 4 Department of Medical Research, Tri-Service General Hospital, Taipei 114, Taiwan; 5 Graduate Institute of Life Sciences, National Defense Medical University, Taipei 114, Taiwan; 6 Medical Affairs Bureau, Ministry of National Defense, No. 325, Sec. 2, Cheng-Kung Road, Neihu, Taipei 114, Taiwan

**Keywords:** burn, inhalation injury, severe hypoxemia, extracorporeal membrane oxygenation, extracorporeal life support

## Abstract

**Background**: Burn patients with severe inhalation injury and refractory hypoxemia are at high risk for cardiorespiratory failure and mortality. Extracorporeal membrane oxygenation (ECMO) has emerged as a potential rescue therapy, but its survival benefits in this population remain uncertain. This study aimed to evaluate the impact of ECMO on mortality in burn patients with severe lung injury, to identify risk factors associated with death, and to analyze causes of rehospitalization among survivors. **Methods**: We conducted a population-based, retrospective cohort study using the Taiwan National Health Insurance Research Database (NHIRD). Burn patients with severe hypoxia requiring mechanical ventilation between 2000 and 2015 were identified. A 0.25-fold propensity score matching was applied based on age, gender, and burn severity. Mortality rates, survival risk factors, and rehospitalization causes were analyzed between ECMO and non-ECMO groups. **Results**: Among 6493 eligible patients, ECMO-treated patients had a hospital mortality rate of 47.09%, compared to 38.71% in the non-ECMO group. Early-phase mortality was higher among ECMO patients (adjusted 1-year mortality HR: 3.19), but survivors demonstrated stable long-term outcomes. Pulmonary complications, cardiac dysfunction, and sepsis were the leading causes of death. Kidney failure and infections were the most common reasons for rehospitalization among survivors. **Conclusions**: This research offers a comprehensive real-world analysis of the effectiveness of ECMO in burn patients. While ECMO does not eliminate early mortality risk, it may provide critical support during acute phase in carefully selected burn patients with severe hypoxemia. Multidisciplinary care and early rehabilitation planning are essential to improve long-term outcomes. Further research is needed to refine patient selection and optimize ECMO strategies in this high-risk population.

## 1. Introduction

For burn patients, mortality rates range from 50% to 80% once severe hypoxemia and ventilator complications occur [1,2]. Severe hypoxemia and mechanical ventilation dependence are common and life-threatening complications in burn patients with extensive inhalation injuries. These conditions, often resulting from thermal airway damage and secondary inflammatory responses, may progress to acute respiratory distress syndrome (ARDS). When standard interventions such as lung-protective ventilation and prone positioning are ineffective, extracorporeal membrane oxygenation (ECMO) may serve as a salvage therapy [3,4]. Although recent studies have reported encouraging outcomes in selected burn patients supported by ECMO, evidence remains inconclusive. Some reports suggest no clear survival benefit, highlighting the need for further stratified evaluation [5,6,7].

Current consensus of ECMO indication from major guidelines and clinical trials, including the ELSO recommendations and the EOLIA trial [8], defines severe hypoxemia as a PaO_2_/FiO_2_ ratio < 50 mmHg for more than 3 h or <80 mmHg for more than 6 h, and severe respiratory acidosis as pH < 7.25 with PaCO_2_ > 60 mmHg for over 6 h. However, despite these established thresholds, there remains no specific guideline or consensus regarding the use of ECMO in burn patients.

Given the complexity of hypoxemia in this population—often related to inhalation injury, airway damage, or systemic inflammatory response—clinical decisions vary across institutions. This lack of standardization highlights the importance of further research focused on ECMO indications and outcomes in burn patients, as addressed in the present study.

This retrospective study using the NHIRD investigates the association between ECMO treatment and survival outcomes, as well as related complications, in patients with burn injuries.

ECMO technology and clinical experience have advanced substantially since the year 2000, with increased application across diverse critical care for patients with refractory cardiac disease, respiratory failure, and burns [6,7]. In Taiwan, ECMO has been reimbursed by the National Health Insurance since 2002 and is widely used in trauma and emergency care [9,10,11]. This study utilizes 16 years of data from the Taiwan National Health Insurance Research Database (NHIRD) to evaluate the impact of ECMO on hospital mortality in burn patients with respiratory failure. Given that conventional strategies are often limited by the need for fluid resuscitation and repeated surgical intervention in this population, ECMO may offer a feasible alternative [12]. We aimed to assess survival outcomes and identify mortality risk factors in burn patients with or without ECMO support.

This retrospective cohort study found that burn patients with hypoxemic respiratory failure who received ECMO had significantly higher risk of mortality. However, specific patients groups, such as adults with respiratory failure, those with inhalation injuries, and individuals with severe burns unresponsive to standard treatments, seem to experience advantages from ECMO therapy [13].

## 2. Material and Methods

### 2.1. Data Sources & Collection, and Ethics Approval

We conducted a population-based retrospective cohort study using Taiwan’s National Health Insurance Research Database (NHIRD). Established in 1995, the program covers over 99% of Taiwan’s residents. Managed by Taiwan’s National Institutes of Health, the NHIRD provides validated, population-representative datasets containing patient demographics, diagnoses, medical interventions, prescriptions, and healthcare costs, including hospital and outpatient reimbursements. Diagnoses in the NHIRD are coded using the International Classification of Diseases 9th Revision, Clinical Modification (ICD-9-CM), and 10th Revision (ICD-10) [14]. All medical records were coded using ICD-9-CM by physicians. The NHIRD, established by Taiwan’s National Health Insurance Administration, contains longitudinal data, including claims for 1,000,000 randomly selected beneficiaries from the Longitudinal Health Insurance Database 2005 (LHID2005). LHID2005 patients showed no significant differences in demographics or insurance-related amounts compared to other NHIRD patients, making it representative of the general population. Patient IDs have been encrypted for privacy, with consistent methods allowing claims linkage within the database. This research was granted an exemption from a comprehensive review by the institutional review board and was conducted with approval from the National Defense Medical University and the Institutional Review Board of the TSGHIRB No. E202216026. OpenAI’s ChatGPT 4o, Word advise, Grammarly were used to assist with grammar and language refinement during manuscript preparation. No scientific content or data analysis was generated by AI. The authors are fully responsible for all content.

### 2.2. Study Design, Enrolled Participants and Study Outcomes

**Figure 1:**
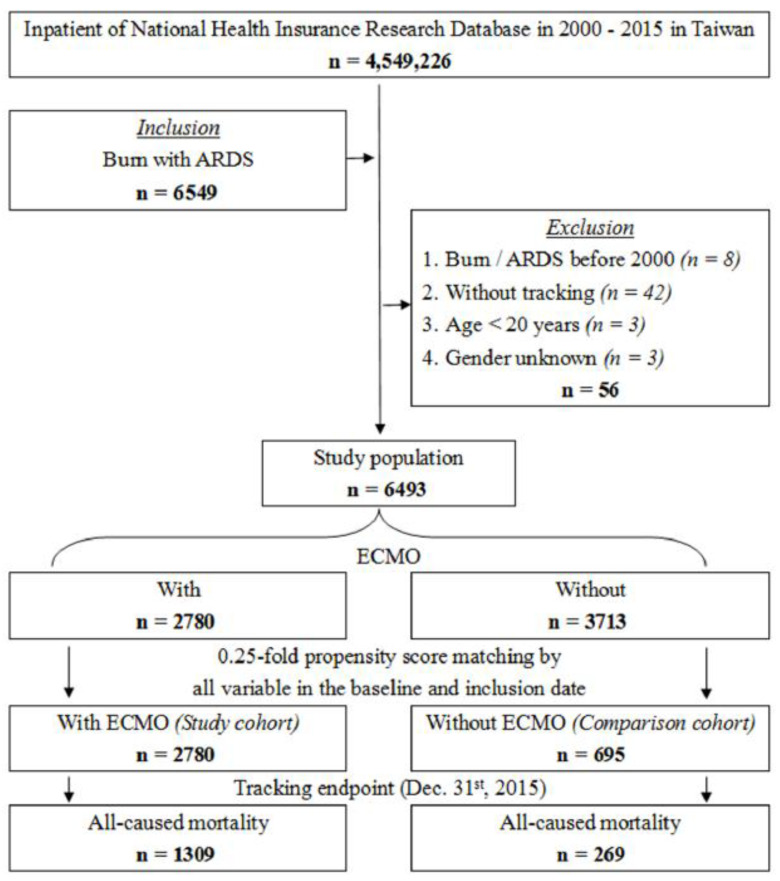
Flowchart of NHIRD.

This study used a matched cohort design, in which we analyzed the risks of mortality among burn patients complicated with lung injury between 1 January 2000 and 31 December 2015. Figure 1 shows the selection criteria was burn patient with ECMO support between 1 January 2000 and 31 December 2015. Of the 4,549,226 individuals recorded in the inpatient data of the database, the study group (Burn patient with severe hypoxia) enrolled 6549 patients diagnosed as burn with severe hypoxia. The exclusion criteria were burn with severe hypoxia before index date (*n* = 8), without tracking (*n* = 42), age less than 20 years old (*n* = 3), and unknown gender (*n* = 3). A total of 6493 patients were identified after applying exclusion criteria. The ECMO group comprised 2780 patients, while the non-ECMO group included 3713 patients prior to matching.

Due to the high clinical heterogeneity within the non-ECMO cohort, 1:1 propensity score matching (PSM) resulted in substantial imbalance in baseline covariates. Therefore, to optimize comparability and reduce confounding, we applied a 0.25-fold nearest-neighbor PSM (without replacement) using the ECMO group as the reference. Matching was performed based on key demographic and clinical variables including age, gender, total body surface area (TBSA), burn depth, and other pre-defined baseline factors shown in Table 1. This yielded a matched cohort of 2780 ECMO patients and 695 non-ECMO patients.

The tracking end point of the study was mortality (ICD-9-CM: E800-E999). Severe hypoxemia was defined ICD-9-CM codes indicating pulmonary insufficiency (518.81–518.82) and the need for mechanical ventilation (procedure codes 96.70–96.72) before the index date. While these codes do not directly represent P/F ratio-based definitions of hypoxemia used in ARDS clinical settings, they serve as validated proxy measures and represent the hypoxia status in NHIRD-based studies. Mortality risk factors were compared, encompassing demographic, burn-related, and facility-related factors, between the study and control groups. These variables were carefully controlled as covariates in this study and were identified using NHIRD claims records. Demographic factors consisted of age and gender, while burn-related factors included injury site, TBSA (Total Body Surface Area), burn extent, mechanical ventilation, aspiration, escharotomy, debridement, tracheotomy, transfusion, hemodialysis, and bacteremia.

The Charlson comorbidity index (CCI) is the most widely used comorbidity index [15]. Comorbidities were assessed using the CCI, which assigns scores based on ICD-9-CM codes from admissions and ambulatory records. The total score indicates the overall comorbidity burden, with higher scores reflecting greater severity [16]. The factors related to the facility comprised geographic location, level of urbanization, and quality of medical care. Cox-regression was utilized to forecast the factors influencing hospital mortality. Additionally, we examined the modified hazard ratio of ECMO within each notable stratified risk factor. The impact of the TBSA and ECMO to the hospital mortality were also examined, and documented the reasons for death in both groups. We also analyzed the impact of ECMO on hospital mortality based on different risk factors, and compared the long-term survival rates between patients who received ECMO and those who did not.

### 2.3. Statistical Analysis

Statistical analysis was conducted using SPSS 25.0 software (SPSS Inc., Chicago, IL, USA), and a significance level of *p* < 0.05 was adopted. Continuous variables were reported as means ± standard deviation and compared using unpaired *t*-tests. Multivariate Cox proportional hazard regression analysis was employed to assess mortality risk, presenting results as hazard ratios (HR) with corresponding 95% confidence intervals (CI). The distinction in mortality risk between the ECMO and non-ECMO groups was determined using the Kaplan–Meier method, and statistical significance was evaluated through the log-rank test.

## 3. Results

### 3.1. Demographics of Enrolled Patients in the Baseline & in the Endpoint

Baseline Characteristics in Table 1. After applying exclusion criteria, 6493 burn patients with severe hypoxemia were identified: 2780 in the ECMO group and 3713 in the non-ECMO group. Following 1:0.25 propensity score matching (age, gender, index date), the final comparison included 2780 ECMO and 695 non-ECMO patients. No significant differences were observed in baseline characteristics such as burn site, TBSA, burn depth, inhalation injury, comorbidities, and hospital level.

### 3.2. In-Hospital Mortality Rate

The in-hospital mortality rate was higher in the ECMO group (47.09%) compared to the non-ECMO group (38.71%, *p* < 0.001). Cox regression showed ECMO use was associated with increased mortality risk (aHR: 1.82, 95% CI: 1.50–2.10, *p* < 0.001). Other significant risk factors included older age (54.97 ± 18.96 vs. 52.73 ± 18.20, *p* < 0.004), male gender, escharotomy, tracheostomy, hemodialysis, transfusion, fluid resuscitation, wound infection, septicemia, organ failure, and higher Charlson Comorbidity Index. In Table 2. All caused mortality in ECMO is 1309 and without ECMO is 269). Cox regression in Table 3 identified ECMO use (aHR 1.82), male gender, older age, greater burn extent, inhalation injury, and multiple complications as significant risk factors for in-hospital mortality in burn patients. 

### 3.3. Effect of ECMO on In-Hospital Mortality in Each Stratified Risk Factor

To establish ECMO as a prominent mortality risk factor, we conducted adjusted Cox regression analyses for each of the significant risk factors mentioned earlier, as presented in Table 4. In relation to gender, age, and burn-related factors. Table 4 shows that taking the without ECMO group as reference, all factors a HR in the ECMO group are significant factors (*p* < 0.001). Among them, patients age greater than 65 (HR 2.38, 95% CI: 1.97–2.75, *p* < 0.001), fluid resuscitation (HR 2.07, 95% CI: 1.71–2.39, *p* < 0.001), hemodialysis (HR 2.16, 95% CI: 1.78–2.49, *p* < 0.001), septicemia (HR 2.11, 95% CI: 1.74–2.44, *p* < 0.001),), and organ failure (aHR: 3.13) had particularly elevated risks. Wound infection (HR 2.96, 95% CI: 2.45–3.42, *p* < 0.001), bacteremia (HR 2.02, 95% CI: 1.67–2.34, *p* < 0.001), shock (HR 2.68, 95% CI: 2.22–3.10, *p* < 0.001) hemorrhage (HR 2.48, 95% CI: 2.05–2.87, *p* < 0.001). The adjusted HR for ECMO in medical center, regional hospital settings was 2.06 (95% CI: 1.71–2.38, *p* < 0.001), 1.19 (95% CI: 0.99–1.38, *p* < 0.001), respectively.

### 3.4. Mortality Risk at Different Terms, All Causes of Mortality & Re-Inpatient Among Survivals

Factors of all-cause mortality among different survival term time by using Cox an analysis of the most common causes of death for such patients is shown in Table 5, Comparing the ECMO group and the non-ECMO group, the survival term defines death within one year as short term mortality, death within 3 years as middle term mortality, and death within 5 years as long term mortality. Short-term (1-year) mortality was significantly higher in ECMO patients (aHR: 3.19, *p* < 0.001). Lung injury, heart failure, sepsis, and renal failure were leading causes of death, with lung injury accounting for 59.74% of deaths in the ECMO group in Table 5.

### 3.5. Rehospitalization Among Survivors

Table 5 presents the analysis of rehospitalization causes among survivors. Among survivors, leading causes of rehospitalization were lung injury (51.94% in ECMO vs. 71.83% in non-ECMO), sepsis (20.0% vs. 29.8%), and kidney failure (12.4% vs. 19.5%), respectively. For total survivors, the most common causes were heart failure at 8.59%, unintentional injuries at 5.11%, multiple organ failure at 1.63%, and tumors at 1.79%.

**Figure 2:**
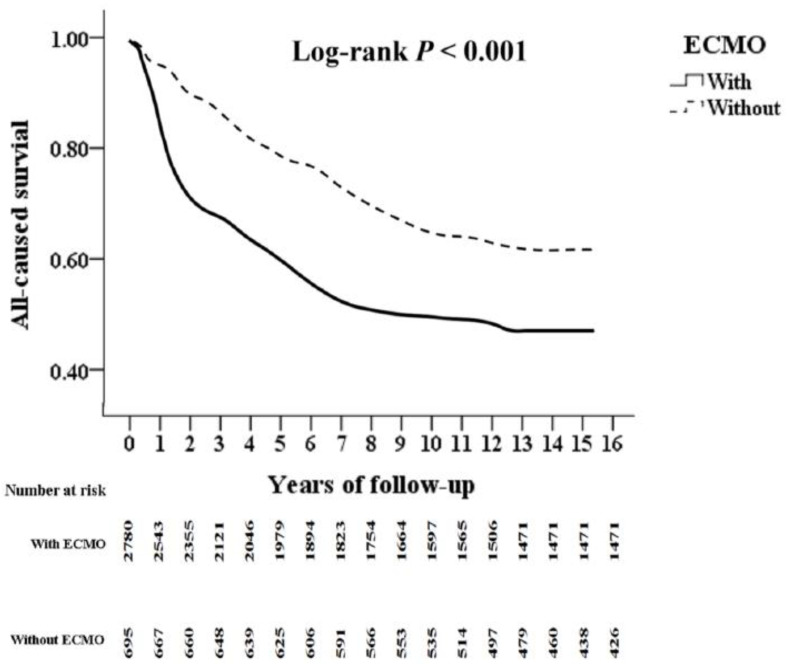
Kaplan–Meier for cumulative survival of all-cause mortality among burn with ARDS inpatients aged 20 and over stratified by ECMO with log-rank test. ECMO: extracorporeal membrane oxygenation.

Kaplan–Meier survival in Figure 2 shows the cumulative survival over time in burn patients with ARDS treated with ECMO. This result shows significantly worse early outcomes for ECMO patients, with survival curves stabilizing after the initial critical phase (log-rank *p* < 0.001). Complete statistical tables are given in the appendix for reference. 

## 4. Discussion

This nationwide population-based cohort study revealed that burn patients with severe hypoxemia who received ECMO treatment had a significantly higher in-hospital mortality rate (47.09%) compared to those who did not receive ECMO (38.71%). The adjusted hazard ratio for ECMO-associated mortality was 1.82, indicating increased early mortality risk. However, long-term outcomes for ECMO survivors stabilized over time. Rehospitalizations among survivors were primarily due to lung injury, kidney failure, and sepsis. These findings underscore the need for careful patient selection, timely ECMO initiation, and multidisciplinary management to optimize outcomes in this high-risk population.

### 4.1. Burn-Related Organ Dysfunction and Cardiopulmonary Failure

Burn-related alveolar-capillary damage increases vascular permeability, resulting in severe dyspnea, hypoxemia, and diffuse pulmonary infiltrates that may progress to ARDS. ECMO has been employed to manage such refractory cases, with reported mortality rates ranging from 48% to 52% in the literature [17]. Johanna et al. reported on 36 pediatric burn patients treated with ECMO, using veno-venous(V-V) and 19 with veno-arterial(V-A) support. The overall survival rate was 53%, comparable to that in pediatric patients requiring ECMO for respiratory failure unrelated to burns [18]. In our study focusing on patients with burn-associated lung injury, the ECMO group had a mortality rate of 47.09%; non-ECMO group exhibited lower mortality rate of 38.71%. These results suggest that while ECMO may not reduce overall mortality, it could serve as a life-saving bridge during acute deterioration. Tiagno S. et al. indicated hyperoxemia following VA-ECMO initiation for cardiogenic shock or cardiac arrest may increase the risk of poor neurological outcomes and mortality. Burn patients are particularly vulnerable to oxidative stress due to systemic inflammation. During ECMO therapy, excessive oxygen exposure may exacerbate cellular injury. Therefore, careful oxygen titration and conservative oxygen targets are essential to avoid additional harm [19].

### 4.2. Cardiac and Pulmonary Failure Are Always the Leading Causes of Early Mortality

Microvascular leakage following burn injury results in plasma volume depletion, increased vascular resistance, and reduced cardiac output. These hemodynamic changes—aggravated by increased blood viscosity and impaired myocardial contractility—often lead to early cardiopulmonary failure [20]. In our cohort, mortality attributable to lung injury and heart failure were higher in the ECMO group (59.74% and 28.72%, respectively) compared to non-ECMO group (18.59% and 8.92%). While acute heart failure may not occur immediately [21], myocardial dysfunction remains a key predictor of early mortality in major burn injuries.

ECMO was correlated with higher dialysis dependence. Patients with extensive burns are also prone to rhabdomyolysis and acute kidney injury, which may necessitate hemodialysis [22]. However, initiating dialysis in unstable patients is often challenging. Although ECMO can support oxygenation and perfusion, it may also contribute to renal stress, increasing the likelihood of dialysis dependence. Conversely, the absence of ECMO support in the non-ECMO group may render patients unable to surpass the critical stage, resulting in higher proportions of mortality attributed to lung damage and heart failure.

Despite these complexities, ECMO has emerged as a salvage therapy in burn patients with refractory hypoxemia. Case reports have demonstrated successful outcomes, even in patients with up to 80% TBSA burns. ECMO enables the safe completion of multiple surgical procedures—including prone-position interventions—while preserving adequate oxygenation [23]. In combination with continuous renal replacement therapy, V-V ECMO has shown promise in cytokine storms and managing sepsis [24]. In a small retrospective series, the survival rate reached 62.5%, comparable to ECMO outcomes in non-burn ARDS patients [25]. These findings support the selective application of ECMO when conventional therapies are insufficient.

### 4.3. ECMO Treatment Gradually Appears After Passing the Critical Phase in the Early Stages

Kaplan–Meier survival analysis (Figure 2) demonstrates that ECMO-treated patients exhibit high early-phase mortality, followed by a plateau in survival rates. This trend indicates that if patients survive the initial critical phase, their long-term outcomes may be comparable to non-ECMO. This trend indicates that if patients survive the initial critical phase, the long-term outcomes may be comparable to those not receiving ECMO. This pattern highlights ECMO’s role in stabilizing severely hypoxic burn patients during the most life-threatening stages. The adjusted hazard ratio for 1-year mortality in the ECMO group was 3.19, indicate higher short-term mortality risk than the non-ECMO group. Despite this, reported mortality rates for ECMO-treated burn patients with severe hypoxia generally fall within the range of 40% to 48%, suggesting potential survival benefit in selected cases [26]. Although some studies report higher mortality in ECMO-supported patients compared to non-ECMO cohorts, these outcomes align with ECMO data in non-burn hypoxemia populations [27]. ECMO may be particularly beneficial for patients with inhalation injury or revised Baux scores exceeding 90 [26]. In one study, favorable outcomes were observed in carefully selected burn patients with ARDS managed with ECMO [28]. However, these patients also face increased complications and prolonged ICU stays, highlighting the need for careful patient selection and post-ECMO care planning [27]. Our findings demonstrate that ECMO-treated burn patients with severe hypoxemia experienced significantly higher in-hospital and short-term mortality, with no evidence of survival benefit across multiple subgroups. While ECMO may provide temporary support in critical deterioration, the absence of long-term outcome improvement and the high complication burden raise concerns regarding its use in this population. These findings underscore the need for rigorous patient selection, cautious interpretation of ECMO benefits, and further investigation through controlled trials to determine whether any subgroup of burn patients may benefit from this therapy.

This study provides population-based evidence from the NHIRD on the association between ECMO use and outcomes in burn patients with hypoxemia. Given the lack of standardized indications in this population, future research should validate our findings through single- and multicenter studies with detailed clinical data, including oxygenation indices and severity scores. Prospective cohorts or registries may further clarify optimal timing, patient selection, and long-term outcomes. Ultimately, developing consensus guidelines for burn patients requiring ECMO will be essential to support clinical decision-making in this critical setting.

## 5. Limitations

This study has several limitations. First, the diagnosis of severe hypoxia relies on ICD-coded data (e.g., acute respiratory failure, pulmonary insufficiency, prolonged mechanical ventilation) rather than clinical assessments, which may only partially capture the extent of pulmonary injury. Burn severity, a major determinant of mortality, could not be fully assessed because coding systems lack precision for variables such as TBSA percentage and standard burn severity scores like the BAUX score. Furthermore, the calculation of mortality risk may be prone to bias due to the death diagnosis-related codes entered by clinicians using ICD-9-CM in the NHIRD.

Second, ECMO initiation criteria may vary across institutions and could not be directly assessed in our dataset. Differences in hospital level and team experience may influence decision thresholds, with high-volume centers more likely to initiate ECMO in complex cases [29]. Such institutional variability may introduce unmeasured confounding and should be considered when interpreting the results.

Third, detailed laboratory, pathological, and treatment parameters—including ECMO mode (V-V vs. V-A), cannulation site, pump speed, and blood flow—were unavailable in the database, limiting in-depth analysis of short- and long-term outcomes.

Fourth, although the NHIRD does not record explicit causes of death, it provides valuable data on severe complications preceding death, allowing indirect assessment of mortality factors.

Although propensity score matching was implemented, residual differences were noted in age and mortality between the ECMO and non-ECMO groups. These discrepancies likely reflect selection biases, as ECMO candidates tend to be younger and more critically ill. We emphasize that standardized mean differences (SMDs) for most covariates were <0.1, indicating acceptable balance. However, residual confounding cannot be excluded and may have influenced observed outcomes.

Despite these limitations, the study offers meaningful real-world insights into the association between ECMO use and mortality risk in burn patients within a national healthcare system.

## 6. Conclusions

This nationwide cohort study found that ECMO use in burn patients with severe hypoxemia was associated with higher early and overall mortality, with no survival benefit observed in any subgroup. Given the complex clinical course, high complication rates, and poor prognosis, although ECMO can provide a vital bridge during acute deterioration, future prospective studies are needed to identify specific patient profiles that may benefit from ECMO and to refine selection criteria accordingly.

## Figures and Tables

**Table 1:** Characteristics of study in the baseline.

ECMO	Total		With		Without		*p*
Variables	*n*	%	*n*	%	*n*	%	
Total	3475		2780	80.00	695	20.00	
Injury site							0.999
Limbs	519	14.94	414	14.89	105	15.11	
Face, head, and neck	1001	28.81	801	28.81	200	28.78	
Trunk	778	22.39	622	22.37	156	22.45	
Multiple sites	1177	33.87	943	33.92	234	33.67	
TBSA							0.999
<10%	130	3.74	104	3.74	26	3.74	
10–29%	594	17.09	475	17.09	119	17.12	
≧30%	1126	32.40	901	32.41	225	32.37	
Unknown	1625	46.76	1300	46.76	325	46.76	
Burn degree							0.990
1–2	379	10.91	303	10.90	76	10.94	
3–4	2279	65.58	1822	65.54	457	65.76	
Unknown	817	23.51	655	23.56	162	23.31	
Inhalation burn							0.957
Without	1177	33.87	941	33.85	236	33.96	
With	2298	66.13	1839	66.15	459	66.04	
Intent							0.909
Unintentional	2933	84.40	2345	84.35	588	84.60	
Intentional	443	12.75	357	12.84	86	12.37	
Unknown	99	2.85	78	2.81	21	3.02	
Catastrophic illness							0.915
Without	1249	35.94	998	35.90	251	36.12	
With	2226	64.06	1782	64.10	444	63.88	
Gender							0.999
Male	1890	54.39	1512	54.39	378	54.39	
Female	1585	45.61	1268	45.61	317	45.61	
Age (years)	48.22 ± 18.73		48.21 ± 18.73		48.25 ± 18.76		0.960
Age group (yrs)							0.996
20–44	1504	43.28	1204	43.31	300	43.17	
45–64	1450	41.73	1159	41.69	291	41.87	
≧65	521	14.99	417	15.00	104	14.96	
Escharotomy							0.972
Without	1323	38.07	1058	38.06	265	38.13	
With	2152	61.93	1722	61.94	430	61.87	
Debridement							0.943
Without	1199	34.50	960	34.53	239	34.39	
With	2276	65.50	1820	65.47	456	65.61	
Tracheostomy							0.986
Without	1936	55.71	1549	55.72	387	55.68	
With	1539	44.29	1231	44.28	308	44.32	
Transfusion							0.941
Without	1034	29.76	828	29.78	206	29.64	
With	2441	70.24	1952	70.22	489	70.36	
Hemodialysis							0.935
Without	2704	77.81	2164	77.84	540	77.70	
With	771	22.19	616	22.16	155	22.30	
Fluid resuscitation							0.917
Without	1376	39.60	1102	39.64	274	39.42	
With	2099	60.40	1678	60.36	421	60.58	
Wound infection							0.804
Without	3370	96.98	2697	97.01	673	96.83	
With	105	3.02	83	2.99	22	3.17	
Septicemia							0.716
Without	3463	99.65	2771	99.68	692	99.57	
With	12	0.35	9	0.32	3	0.43	
Bacteremia							0.360
Without	3473	99.94	2779	99.96	694	99.86	
With	2	0.06	1	0.04	1	0.14	
Shock							0.633
Without	3468	99.80	2775	99.82	693	99.71	
With	7	0.20	5	0.18	2	0.29	
Hemorrhage							0.802
Without	3471	99.88	2777	99.89	694	99.86	
With	4	0.12	3	0.11	1	0.14	
DIC							0.999
Without	3470	99.86	2776	99.86	694	99.86	
With	5	0.14	4	0.14	1	0.14	
Organ failure							0.664
Without	3467	99.77	2774	99.78	693	99.71	
With	8	0.23	6	0.22	2	0.29	
CCI_R	1.21 ± 1.73		1.21 ± 1.74		1.19 ± 1.71		0.786
Level of care							0.827
Hospital center	2480	71.37	1982	71.29	498	71.65	
Regional hospital	992	28.55	796	28.63	196	28.20	
Local hospital	3	0.09	2	0.07	1	0.14	

*p*: Chi-square/Fisher exact test on category variables and *t*-test on continue variables.

**Table 2:** Characteristics of study in the endpoint.

ECMO	Total		With		Without		*p*
Variables	*n*	%	*n*	%	*n*	%	
Total	3475		2780	80.00	695	20.00	
All-cause mortality							<0.001
Survival	1897	54.59	1471	52.91	426	61.29	
Death	1578	45.41	1309	47.09	269	38.71	
Injury site							0.999
Limbs	519	14.94	414	14.89	105	15.11	
Face, head, and neck	1001	28.81	801	28.81	200	28.78	
Trunk	778	22.39	622	22.37	156	22.45	
Multiple specified sites	1177	33.87	943	33.92	234	33.67	
TBSA							0.999
<10%	130	3.74	104	3.74	26	3.74	
10–29%	594	17.09	475	17.09	119	17.12	
≧30%	1126	32.40	901	32.41	225	32.37	
Unknown	1625	46.76	1300	46.76	325	46.76	
Burn degree							0.990
1–2	379	10.91	303	10.90	76	10.94	
3–4	2279	65.58	1822	65.54	457	65.76	
Unknown	817	23.51	655	23.56	162	23.31	
Inhalation burn							0.957
Without	1177	33.87	941	33.85	236	33.96	
With	2298	66.13	1839	66.15	459	66.04	
Intent							0.909
Unintentional	2933	84.40	2345	84.35	588	84.60	
Intentional	443	12.75	357	12.84	86	12.37	
Unknown	99	2.85	78	2.81	21	3.02	
Gender							0.999
Male	1890	54.39	1512	54.39	378	54.39	
Female	1585	45.61	1268	45.61	317	45.61	
Age (yrs)	53.18 ± 18.37		52.73 ± 18.20		54.97 ± 18.96		0.004
Age group (yrs)							0.101
20–44	1453	41.81	1172	42.16	281	40.43	
45–64	1413	40.66	1140	41.01	273	39.28	
≧65	609	17.53	468	16.83	141	20.29	
Escharotomy							0.306
Without	1262	36.32	998	35.90	264	37.99	
With	2213	63.68	1782	64.10	431	62.01	
Debridement							0.858
Without	1200	34.53	962	34.60	238	34.24	
With	2275	65.47	1818	65.40	457	65.76	
Tracheostomy							0.918
Without	1936	55.71	1550	55.76	386	55.54	
With	1539	44.29	1230	44.24	309	44.46	
Transfusion							0.473
Without	1059	30.47	855	30.76	204	29.35	
With	2416	69.53	1925	69.24	491	70.65	
Hemodialysis							0.142
Without	2605	74.96	2069	74.42	536	77.12	
With	870	25.04	711	25.58	159	22.88	
Fluid resuscitation							0.444
Without	1316	37.87	1044	37.55	272	39.14	
With	2159	62.13	1736	62.45	423	60.86	
Wound infection							0.471
Without	3320	95.54	2652	95.40	668	96.12	
With	155	4.46	128	4.60	27	3.88	
Septicemia							0.350
Without	3385	97.41	2704	97.27	681	97.99	
With	90	2.59	76	2.73	14	2.01	
Bacteremia							0.867
Without	3466	99.74	2773	99.75	693	99.71	
With	9	0.26	7	0.25	2	0.29	
Shock							0.893
Without	3461	99.60	2769	99.60	692	99.57	
With	14	0.40	11	0.40	3	0.43	
Hemorrhage							0.999
Without	3465	99.71	2772	99.71	693	99.71	
With	10	0.29	8	0.29	2	0.29	
DIC							0.595
Without	3467	99.77	2773	99.75	694	99.86	
With	8	0.23	7	0.25	1	0.14	
Organ failure							0.055
Without	2701	77.73	2142	77.05	559	80.43	
With	774	22.27	638	22.95	136	19.57	
CCI_R	1.34 ± 1.95		1.35 ± 1.97		1.28 ± 1.86		0.397
Level of care							0.463
Hospital center	2471	71.11	1977	71.12	494	71.08	
Regional hospital	996	28.66	798	28.71	198	28.49	
Local hospital	8	0.23	5	0.18	3	0.43	

ECMO: extracorporeal membrane oxygenation; TBSA: total body surface area; *p*: Chi-square/Fisher exact test on category variables and *t*-test on continue variables; DIC: disseminated idiopathy coagulopathy.

**Table 3:** Factors of mortality by using Cox regression.

Variables	Crude HR	95% CI	95% CI	*p*	aHR	95% CI	95% CI	*p*
ECMO								
Without	Reference				Reference			
With	2.26	1.91	2.53	<0.001	1.82	1.50	2.10	<0.001
Injury site								
Limbs	Reference				Reference			
Face, head, and neck	1.46	1.31	1.55	<0.001	1.44	1.31	1.55	<0.001
Trunk	1.39	1.24	1.50	<0.001	1.38	1.22	1.50	<0.001
Multiple sites	1.53	1.41	1.62	<0.001	1.52	1.40	1.62	<0.001
TBSA								
<10%	Reference				Reference			
10–29%	1.40	1.37	1.47	<0.001	1.39	1.36	1.46	<0.001
≧30%	1.48	1.39	1.53	<0.001	1.47	1.38	1.52	<0.001
Unknown	1.32	1.21	1.39	<0.001	1.31	1.21	1.38	<0.001
Burn degree								
1–2	Reference				Reference			
3–4	1.53	1.41	1.63	<0.001	1.52	1.40	1.62	<0.001
Unknown	1.14	0.77	1.32	0.228	1.13	0.77	1.31	0.235
Inhalation burn								
Without	Reference				Reference			
With	1.66	1.51	1.77	<0.001	1.64	1.50	1.75	<0.001
Intent								
Unintentional	Reference				Reference			
Intentional	1.58	1.42	1.68	<0.001	1.57	1.40	1.66	<0.001
Unknown	1.24	1.13	1.60	<0.001	1.25	1.20	1.59	<0.001
Gender								
Male	1.71	1.60	1.80	<0.001	1.59	1.44	1.63	<0.001
Female	Reference				Reference			
Age group (yrs)								
20–44	Reference				Reference			
45–64	1.62	1.51	1.66	<0.001	1.60	1.46	1.64	<0.001
≧65	1.77	1.67	1.81	<0.001	1.73	1.65	1.80	<0.001
Escharotomy								
Without	Reference				Reference			
With	3.15	2.67	3.80	<0.001	1.95	1.73	2.03	<0.001
Debridement								
Without	Reference				Reference			
With	2.83	2.28	2.90	<0.001	1.78	1.62	1.80	<0.001
Tracheostomy								
Without	Reference				Reference			
With	3.28	2.85	3.64	<0.001	1.82	1.65	1.93	<0.001
Transfusion								
Without	Reference				Reference			
With	3.19	2.38	3.44	<0.001	1.95	1.83	2.13	<0.001
Hemodialysis								
Without	Reference				Reference			
With	2.83	2.21	3.16	<0.001	1.74	1.63	1.80	<0.001
Fluid resuscitation								
Without	Reference				Reference			
With	2.89	2.14	3.21	<0.001	1.91	1.78	2.00	<0.001
Wound infection								
Without	Reference				Reference			
With	1.75	1.60	1.86	<0.001	1.60	1.39	1.78	<0.001
Septicemia								
Without	Reference				Reference			
With	2.81	2.37	3.17	<0.001	1.72	1.59	1.85	<0.001
Bacteremia								
Without	Reference				Reference			
With	2.70	2.33	3.14	<0.001	1.98	1.78	2.15	<0.001
Shock								
Without	Reference				Reference			
With	2.13	1.71	2.36	<0.001	1.58	1.41	1.69	<0.001
Hemorrhage								
Without	Reference				Reference			
With	1.82	1.60	2.23	<0.001	1.46	1.39	1.50	<0.001
DIC								
Without	Reference				Reference			
With	3.19	2.61	4.17	<0.001	1.86	1.72	2.10	<0.001
Organ failure								
Without	Reference				Reference			
With	2.06	1.67	2.29	<0.001	1.70	1.43	1.76	<0.001
CCI_R	1.58	1.45	1.65	<0.001	1.47	1.37	1.58	<0.001
Level of care								
Hospital center	2.37	2.03	2.63	<0.001	2.07	1.77	2.28	<0.001
Regional hospital	2.20	1.78	2.41	<0.001	1.82	1.50	2.06	<0.001
Local hospital	Reference				Reference			

Ahr = adjusted HR—adjusted variables listed in the table; CI = confidence interval.

**Table 4:** Factors of all-cause mortality stratified by variables listed in the table by using Cox regression and Bonferroni correction for multiple comparisons.

ECMO	With			Without (Reference)			With vs. Without (Reference)			
Strarified	Events	PYs	Rate (per 10^5^ PYs)	Events	PYs	Rate (per 10^5^ PYs)	aHR	95% CI	95% CI	*p*
Total	1309	22,463	5827	269	5619	4787	1.82	1.50	2.10	<0.001
Gender										
Male	693	12,244	5660	131	3057	4285	1.97	1.63	2.28	<0.001
Female	616	10,219	6028	138	2562	5386	1.67	1.38	1.93	<0.001
Age group										
20–44	520	9472	5490	119	2271	5239	1.56	1.29	1.81	<0.001
45–64	567	9159	6191	101	1998	5054	1.83	1.51	2.11	<0.001
≧65	222	3832	5793	49	1349	3631	2.38	1.97	2.75	<0.001
Escharotomy										
Without	556	8051	6906	125	2021	6186	1.67	1.38	1.93	<0.001
With	753	14,412	5225	144	3598	4002	1.95	1.61	2.25	<0.001
Debridement										
Without	653	7677	8506	117	1541	7594	1.67	1.38	1.93	<0.001
With	656	14,787	4436	152	4078	3727	1.78	1.47	2.06	<0.001
Tracheostomy										
Without	692	12,397	5582	122	2635	4630	1.80	1.49	2.08	<0.001
With	617	10,066	6130	147	2984	4926	1.86	1.54	2.15	<0.001
Transfusion										
Without	532	6516	8164	126	1650	7635	1.60	1.32	1.85	<0.001
With	777	15,947	4872	143	3969	3603	2.02	1.67	2.33	<0.001
Hemodialysis										
Without	858	16,763	5118	200	4360	4587	1.67	1.38	1.93	<0.001
With	451	5700	7912	69	1259	5481	2.16	1.78	2.49	<0.001
Fluid resuscitation										
Without	382	8423	4535	106	2200	4818	1.41	1.16	1.63	<0.001
With	927	14,040	6603	163	3419	4768	2.07	1.71	2.39	<0.001
Wound infection										
Without	1221	21,491	5682	259	5400	4796	1.77	1.46	2.05	<0.001
With	88	972	9051	10	219	4568	2.96	2.45	3.42	<0.001
Septicemia										
Without	1291	22,218	5811	263	5504	4779	1.82	1.50	2.10	<0.001
With	18	245	7351	6	115	5206	2.11	1.74	2.44	<0.001
Bacteremia										
Without	1305	22,401	5826	268	5603	4787	1.82	1.50	2.10	<0.001
With	4	62	6477	1	16	4784	2.02	1.67	2.34	<0.001
Shock										
Without	1303	22,382	5822	268	5595	4790	1.81	1.50	2.10	<0.001
With	6	81	7380	1	24	4107	2.68	2.22	3.10	<0.001
Hemorrhage										
Without	1304	22,401	5821	268	5603	4787	1.82	1.50	2.10	<0.001
With	5	62	8093	1	16	4869	2.48	2.05	2.87	<0.001
DIC										
Without	1305	22,417	5821	269	5610	4788	1.82	1.50	2.10	<0.001
With	4	46	8713	0	9	4388	∞	-	-	0.999
Organ failure										
Without	794	17,462	4547	215	4519	4757	1.43	1.18	1.65	<0.001
With	515	5001	10,298	54	1100	4911	3.13	2.59	3.62	<0.001
Level of care										
Center	1063	15,925	6675	193	3993	4833	2.06	1.71	2.38	<0.001
Regional	245	6462	3791	76	1601	4748	1.19	0.99	1.38	<0.001
Local	1	76	1317	0	25	0	∞	-	-	0.999

PYs = person-years; aHR = adjusted hazard ratio—adjusted for the variables listed in Table 3; CI = confidence interval.

**Table 5:** Mortality risk at different terms, all causes of mortality and re-inpatient among survivals.

ECMO	With vs. Without (Reference)							
Survival Term	aHR			95% CI		95% CI		*p*
**Overall**	1.82			1.50		2.10		<0.001
1-year mortality (Short-term mortality)	3.19			2.34		3.48		<0.001
3-year mortality (Middle-term mortality)	1.61			1.08		1.98		0.010
5-year mortality (Long-term mortality)	1.82			1.42		2.04		<0.001
**All causes of mortality**								
**ECMO**	**Total** (*n* = 3475)		**With** (*n* = 2780)				**Without** (*n* = 695)	
**Multiple causes**	***n***	**%**	***n***		**%**		***n***	**%**
All-cause mortality	1578		1309				269	
Severe hypoxemia/Pneumonia	832	52.72	782		59.74		50	18.59
Heart failure/Cardiac arrest	400	25.35	376		28.72		24	8.92
Septicemia/Bacteremia	240	15.21	230		17.57		10	3.72
Kidney failure/ESRD/Hemodialysis	221	14.01	212		16.20		9	3.35
Multiple organ failure	159	10.08	151		11.54		8	2.97
Unintentional injury	83	5.26	78		5.96		5	1.86
Suicide	6	0.38	5		0.38		1	0.37
Tumors	35	2.22	33		2.52		2	0.74
Burn	78	4.94	74		5.65		4	1.49
Others	65	4.12	62		4.74		3	1.12
**All causes of re-inpatient among survivals**								
**ECMO**	**Total** (*n* = 3475)		**With** (*n* = 2780)				**Without** (*n* = 695)	
**Multiple causes**	***n***	**%**	***n***		**%**		***n***	**%**
All-cause survival re-inpatient	1897		1471				426	
Severe hypoxemia/Pneumonia	1070	56.40	764		51.94		306	71.83
Heart failure/Cardiac arrest	163	8.59	115		7.82		48	11.27
Septicemia/Bacteremia	421	22.19	294		19.99		127	29.81
Kidney failure/ESRD/Hemodialysis	266	14.02	183		12.44		83	19.48
Multiple organ failure	31	1.63	20		1.36		11	2.58
Unintentional injury	97	5.11	72		4.89		25	5.87
Suicide	1	0.05	1		0.07		0	0.00
Tumors	34	1.79	28		1.90		6	1.41
Burn	118	6.22	83		5.64		35	8.22
Others	73	3.85	55		3.74		18	4.23

## Data Availability

The data presented in this study are available from the NHIRD, maintained by the Health and Welfare Data Science Center (HWDC), Ministry of Health and Welfare, Taiwan. The data is not publicly available due to legal and ethical restrictions. Access to the NHIRD is restricted to qualified researchers who meet the criteria for access and submit a formal application through the HWDC portal: https://dep.mohw.gov.tw/DOS/np-2497-113.html (accessed on 1 January 2022). The data that support the findings of this study are available from Taiwan NHIRD, but restrictions apply to the availability of these data, which were used under license for the current study, and so are not publicly available. Data are, however, available from the authors upon reasonable request and with permission of Taiwan NHIRD.
